# Pernicious Anemia: A Rare Case of Vitamin B12 Deficiency With Hyperpigmentation and Pancytopenia

**DOI:** 10.1155/carm/1262770

**Published:** 2026-09-08

**Authors:** Emmanuel Jojo Aryee, Helen Agyei-Yeboah

**Affiliations:** ^1^ Department of Medicine, Greater Accra Regional Hospital, Accra, Ghana, duhs.edu.pk

**Keywords:** hyperpigmentation, intrinsic factor, pancytopenia, pernicious anemia, vitamin B12 deficiency

## Abstract

The clinical mimicry of vitamin B12 deficiency can often delay its recognition and treatment. Pernicious anemia remains among its major clinical causes. We report the case of a 32‐year‐old male who presented with symptoms of progressive darkened skin, fatigue, general weakness, and neurological symptoms, with examination notable of severe conjunctival pallor and prominent hyperpigmentation of the buccal mucosa, hands, and feet. Investigations revealed pancytopenia with critically low serum vitamin B12. The presence of serum intrinsic factor antibodies confirmed the diagnosis of pernicious anemia. Parenteral vitamin B12 supplementation resulted in rapid hematological improvement and resolution of hyperpigmentation. This case underscores that hyperpigmentation, pancytopenia, and neurological symptoms should prompt a higher index of suspicion for vitamin B12 deficiency to prevent severe hematologic and neurologic complications with closer attention given to follow‐up.

## 1. Introduction

Vitamin B12 plays a prominent role in hematopoiesis, deoxyribonucleic acid, ribonucleic acid synthesis, and myelination of neurons [1]. It is solely derived from dietary sources such as meat, poultry, shellfish, and dairy products [2]. Its absorption in the terminal ileum is essentially dependent on its complex with intrinsic factor which is produced by the gastric parietal cells. Pernicious anemia, an autoimmune condition, occurs when intrinsic factor antibodies (IFAbs) prevent this absorption from occurring by inhibiting the binding of vitamin B12 to intrinsic factor [1, 2].

Total body stores of vitamin B12, averaging between 3 and 5 mg, are depleted within 5–10 years once there is a significant reduction in the intake of vitamin B12‐rich foods [3]. Symptoms of vitamin B12 deficiency begin to manifest when serum levels fall significantly below 200 pg/mL (148 pmol/L) [4]. Etiology for vitamin B12 deficiency is put into four main categories: autoimmune (e.g., pernicious anemia), malabsorption (e.g., inflammatory bowel disease, celiac disease, surgical gastrectomy and ileal resection, *Diphyllobothrium latum* infestation, and pancreatic insufficiency), dietary insufficiency (e.g., vegan diets), and due to toxins or medications (e.g., proton pump inhibitors, and metformin) [2]. A comprehensive evaluation and a high index of suspicion are therefore necessary for early diagnosis and management.

Clinically, vitamin B12 deficiency can manifest with a myriad of hematologic, neuropsychiatric, and digestive symptoms [2]. Although rare, cutaneous manifestations may also occur, including hyperpigmentation of the extremities, particularly over the dorsum of the hands and feet [5, 6]. Here, we report an unusual case of vitamin B12 deficiency presenting with both hyperpigmentation and pancytopenia secondary to pernicious anemia. Diagnosis was made following a workup indicating reduced serum vitamin B12 and elevated levels of serum IFAbs.

## 2. Case Report

A 32‐year‐old male patient was referred to our emergency department by a family medicine physician on account of pancytopenia with severe anemia of 5.8 g/dL (reference: 13.0–18.0 g/dL). He presented with a 2‐year medical history of progressive generalized hyperpigmentation, mainly of the hands and feet which was associated with easy fatiguability, loss of appetite, palpitations, headache, dizziness, and weight loss. He also experienced paresthesia in both hands and frequent episodes of forgetfulness over 6 months prior to his presentation. There was no gait abnormality, seizures, tremors, or paralysis. His past medical history was unremarkable. He had been prescribed hematinics (iron and folic acid supplements) on earlier presentation of his symptoms at a clinic but had seen no improvement. He has no history of smoking, alcohol, and illicit drug use. He also had no dietary restrictions and consumed meat on a weekly basis. He had no family history of any similar illness and had no prior history of blood transfusions. He had a 4‐year history working on electrical transformers, which involved direct exposure to hazardous materials including copper, silicon, mineral oil, and other potential chemical contaminants.

**FIGURE 1:**
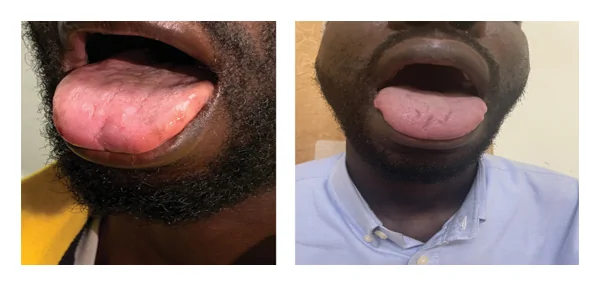
Tongue at initial presentation and 2 weeks posttreatment, showing a smooth, beefy‐red tongue with loss of lingual papillae (glossitis) of the tongue with mild improvement following vitamin B12 supplementation.

**FIGURE 2:**
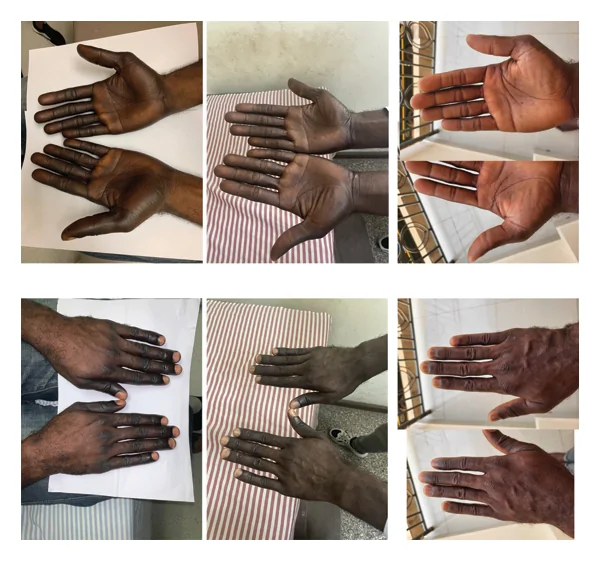
Hands (palms and dorsum) at initial presentation, 2 weeks, and 4 weeks posttreatment, showing uniform hyperpigmentation at baseline with progressive lightening of the pigmentation following parenteral vitamin B12 therapy.

**FIGURE 3:**
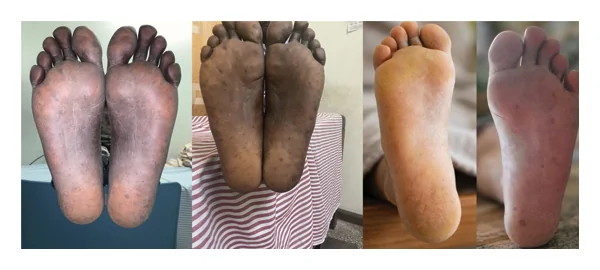
Feet (plantar surface) at initial presentation, 2 weeks, and 4 weeks posttreatment, demonstrating hyperpigmented macules on the soles at baseline with gradual fading over the course of treatment.

**FIGURE 4:**
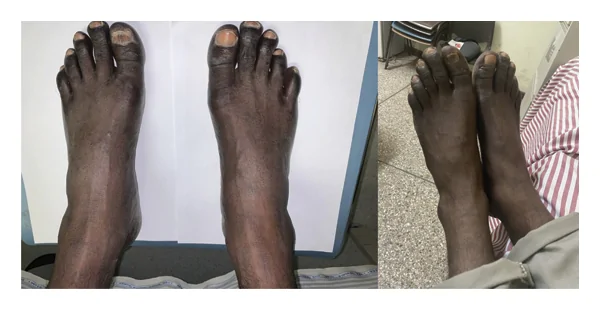
Feet (dorsal surface) at initial presentation and 2 weeks posttreatment, showing hyperpigmentation with mild improvement after two weeks of parenteral vitamin B12 therapy.

On initial physical examination, the patient looked chronically unwell and appeared very weak. He had severe conjunctival pallor and mild scleral icterus. Oral examination showed patchy areas of hyperpigmentation on the buccal mucosa with the tongue having a beefy‐red appearance and loss of lingual papillae consistent with glossitis (Figure 1). He had silky, easily pluckable scalp hair. There was no evidence of regional lymphadenopathy on palpation of the cervical, axillary, or inguinal lymph nodes. Dermal inspection showed uniform hyperpigmentation involving both palms, knuckles of the fingers, dorsum of both feet, and plantar surface of the feet, alongside a few hyperpigmented macules on the upper limbs, torso, and soles of both feet (Figures 2–4). Cardiovascular, respiratory, gastrointestinal, and neurological system examinations were otherwise unremarkable. The mini‐mental state assessment conducted as part of neurological examination was also normal.

Accompanying laboratory results showed severe anemia of 5.8 g/dL (mean corpuscular hemoglobin [Hb] 33.7 pg, mean corpuscular volume 97 fL, and red cell distribution width of 21), leukopenia of 0.91 × 10^9^/L, thrombocytopenia of 135 × 10^3^/L, and reticulocytes count of 0.18% (reference range: 0.5%–2.0%). His serum vitamin B12 level was low at 65 pmol/L (reference range: 133–675 pmol/L), and iron studies indicated normal serum iron (29.8 μmol/L) and serum ferritin levels (329.5 ng/mL), with low serum transferrin (18.9 μmol/L) and high transferrin saturation of 78.8%. His liver function tests were deranged, with an indirect hyperbilirubinemia (total bilirubin 64 μmol/L and direct bilirubin of 10 μmol/L) and transaminitis: aspartate transaminase 220 U/L, alanine transaminase 146 U/L, alkaline phosphatase 197 U/L, and gamma‐glutamyl transferase 182 U/L. Stool for occult blood was positive on a guaiac‐based fecal occult blood test (gFOBT). Serum folate level, thyroid function tests were normal, and the retro screen was nonreactive.

Based on the clinical presentation and initial accompanying investigations, a diagnosis of pancytopenia secondary to a vitamin B12 deficiency was made, with differentials of possible hematological malignancy, Addison’s disease, heavy metal poisoning, and connective tissue disease.

Patient was then admitted to the facility. Further laboratory investigations, namely: repeat complete blood count (CBC), peripheral blood film, fecal immunochemical test (FIT), renal function tests, liver function tests, serum uric acid, urinalysis, adrenocorticotropic hormone (ACTH), serum IFAb test, extractable nuclear antigen (ENA) panel, antinuclear antibody (ANA) profile, erythrocyte sedimentation rate (ESR), and imaging studies (including computed tomography [CT] scan of the abdomen and endoscopy), were requested to identify the cause of the vitamin B12 deficiency and to rule out the differential diagnoses. Treatment was initiated (after blood draws) with transfusion of two units of whole blood and parenteral treatment with vitamin B12 (1000 µg thrice a week for 4 weeks, then once every month for life).

Results from the peripheral blood smear showed anisocytosis, macroovalocytes, hypersegmented neutrophils, neutropenia and mild thrombocytopenia with no clumps seen. Serum IFAb was positive, with a value of 36.36 AU/mL (reference range: 1.2–1.5 AU/mL). Auto antibodies for gastric parietal cell, Scl‐70, Jo‐1, SM, SSA, SSB, RNP, CENP, and antinuclear factor, proliferating cell nuclear antigen were negative.

ESR and lactate dehydrogenase (LDH) were both elevated with values of 122 mm/hr (reference range: 0–15 mm/hr) and 5576 IU/L (reference range: 135–225 IU/L), respectively. The FIT was negative, indicating a previous false positive stool test, likely explained by hematinic taken.

Other tests revealed normal results: uric acid 0.31 mmol/L, negative stool test for *Helicobacter pylori*, normal urinalysis, normal renal function, serum copper 21.7 µmol/L, urinary free copper 0.56 µmol/L, serum lead 0.56 µg/dL, serum ceruloplasmin 31 mg/dL, and ACTH were all within normal ranges. Viral hepatitis serology was negative.

A chest X‐ray was also normal. Additional imaging, including a CT scan of the abdomen as well as both upper and lower gastrointestinal endoscopies, was planned to exclude an underlying gastrointestinal malignancy were deferred due to financial constraints of the patient and scheduled for a later date on an outpatient basis.

A diagnosis of vitamin B12 deficiency secondary to pernicious anemia was made and management continued with parenteral vitamin B12 treatment. Posttransfusion CBC showed an improvement in the Hb level (7.5 g/dL) and white blood cell (WBC) count (2.1 × 10^9^/L). Patient was discharged after a week of admission to be followed up on outpatient basis.

After two weeks of the intramuscular vitamin B12 injections, there was significant clinical improvement, with a return of normal CBC indices (Hb of 11 g/dL, WBC count of 4.8 × 10^9^/L, and platelet count of 390 × 10^3^/L) (Table 1). Progressive resolution of hyperpigmentation was noted on the hands and soles of the feet (Figures 1–4). Serum vitamin B12 levels were elevated to 8664pmol/L (reference ranges: 133–675 pmol/L). Given the markedly elevated level of vitamin B12, intramuscular injections were held for a two‐week period as a precautionary clinical decision, with consideration for vitamin B12 toxicity (although no symptoms were reported in the period). Following this interval, injections were resumed and continued as monthly maintenance therapy as part of the lifelong replacement regimen.

**TABLE 1:** Complete blood count and serum vitamin B12 results.

Parameters	Pretransfusion	Posttransfusion (2 days)	2 weeks post‐IM vitamin B12 injections	Reference range
Hb (g/dL)	5.8	7.5	11.0	13.0–18.0
MCV (fL)	97	90	89	76–99
MCH (pg)	33.7	30.4	28.2	26–33
WBC (× 10^9^/L)	2.7	2.1	4.8	4.0–12.0
Platelets (× 10^3^/μL)	135	65	390	150–450
Serum vitamin B12 (pmol/L)	65	—	8664	133–675

*Note:* Hb = hemoglobin.

Abbreviations: MCH = mean corpuscular hemoglobin, MCV = mean corpuscular volume, WBC = white blood cells.

## 3. Discussion

Cutaneous hyperpigmentation occurring with pancytopenia should prompt early medical consideration of vitamin B12 deficiency as a differential diagnosis, as it is one of the principal treatable causes of combined hematological, neurological, and dermatological pathology [7, 8].

The reported prevalence of cutaneous hyperpigmentation among vitamin B12‐deficient patients varies from 5% to 10% across case series in literature and is noted to be more conspicuous in individuals with darker skin tones (Fitzpatrick skin types V‐VI) [3, 5, 6, 8]. Hyperpigmentation occurs when B12 deficiency causes a reduction in intracellular glutathione levels, which inhibits tyrosinase activity and leads to accelerated synthesis of eumelanin in highly reactive melanocytes. This results in hypermelanosis that is significantly more pronounced and clinically conspicuous in individuals with darker baseline skin tones (due to the presence of highly reactive melanocytes). Published case series therefore highlight disproportionate representation among patients of African, South Asian, and Middle Eastern descent [3, 5, 7–9]. This demographic phenotypic variation poses diagnostic challenges in sub‐Saharan African settings, where misattributing cutaneous hyperpigmentation to postinflammatory changes, adrenal insufficiency (Addison’s disease), or physiological changes potentially delays diagnosis and treatment. A case reported from Ghana by Awindago et al. described reversible hyperpigmentation in vitamin B12 deficiency that mimicked Addison’s disease [7]. Pigmentation in B12 deficiency characteristically involves the dorsa of the hands and feet, palmar creases, oral mucosa, and nail beds, as in our patient, and resolves gradually with replacement therapy.

Pernicious anemia is an advanced manifestation of autoimmune gastritis, which occurs when there is T‐cell‐mediated destruction of gastric parietal cells with consequent intrinsic factor deficiency [10, 11]. This intrinsic factor deficiency disrupts absorption of vitamin B12 in the terminal ileum, leading to vitamin B12 deficiency [11]. Pancytopenia occurs consequent to vitamin B12 deficiency due to impaired DNA synthesis. Cobalamin, as an essential cofactor in DNA synthesis, is required to convert deoxyuridine monophosphate to deoxythymidine monophosphate, and in its absence, all rapidly dividing hematopoietic progenitor cells in the bone marrow are affected. This produces the megaloblastic and ineffective hematopoiesis across all three cell lines in the bone marrow, mimicking aplastic anemia and hematological malignancy, as was encountered in the differential diagnosis of our patient, highlighting the importance of including vitamin B12 deficiency in the workup of unexplained pancytopenia, particularly in resource‐limited settings where bone marrow biopsy may not be immediately accessible.

A diagnosis of pernicious anemia is therefore made when there is low serum vitamin B12, megaloblastic anemia, and positive titers for antibodies against intrinsic factor or gastric parietal cells, as was seen in this case report [10]. These antibodies are present in 50%–70% of patients with pernicious anemia [10, 12]. Parietal cell antibodies (PCA) have been noted to be more sensitive (80%–90%) but less specific (50%–70%) for autoimmune gastritis, and their titers may decline, or they may become undetectable in advanced disease as the parietal cell mass progressively gets destroyed. In sharp contrast, not only are IFAb highly specific (95%–100%) though less sensitive (50%–70%) for pernicious anemia, but they also persist for extended periods. Pernicious anemia is prevalent in patients over 30 years with no gender predisposition [8]. Its prevalence in the Ghanaian population is, however, yet to be determined. Patients with pernicious anemia usually present with vague symptoms initially, but as the disease progresses, more defined and serious complications arise, usually involving the hematological, neurological systems and, in our case, the dermatological system [12].

Serum methylmalonic acid (MMA) and homocysteine are also sensitive functional biomarkers of vitamin B12 deficiency and are particularly helpful for subclinical B12 deficiency or the early phase of the condition. In vitamin B12 deficiency, MMA accumulates due to impaired conversion of methylmalonyl‐CoA to succinyl‐CoA, while homocysteine rises due to defective remethylation to methionine. Both are elevated even before overt hematological changes manifest, hence their usefulness in cases with subclinical B12 deficiency. In our patient, MMA and homocysteine levels were not formally reported; their inclusion in future cases would strengthen the diagnostic evidence base and help clarify the functional severity of deficiency.

In severe cases of pernicious anemia, derangements in the liver function evidenced by elevated liver enzymes and LDH have been identified [13]. These abnormalities are suggestive of hypoxic hepatitis that can occur when there is significant reduction in oxygen delivery to the liver due to severe chronic anemia [13]. Prognosis has been reported to be poor; however, as seen in our patient, there was significant clinical improvement after initiating treatment. Other causes of hepatocellular injury should be actively excluded before attributing transaminitis to hypoxic hepatitis [13]. In our patient, viral hepatitis was excluded serologically, and abdominal ultrasonography showed no focal hepatic lesion or abnormality.

Treatment of pernicious anemia consists of lifelong parenteral replacement of vitamin B12 [11]. A typical regimen is 1000 μg administered two or three times weekly for the first one to 4 weeks to replenish body cobalamin stores, followed by a maintenance dose everyone to 3 months through a preferred parenteral route [11]. Normalization of CBC indices is usually seen after 2–4 weeks of initiating treatment [10], while neurological recovery follows a slower trajectory of over 3 months to a year, and a minority of patients may be left with residual deficit, particularly when treatment is delayed [14]. Although parenteral vitamin B12 has a wide safety margin and no established toxicity threshold that would mandate withholding maintenance therapy, a brief precautionary pause was clinically elected in this patient given the markedly supraphysiological level recorded; no features of toxicity were observed, and maintenance therapy was subsequently continued without interruption to hematological or dermatological recovery.

A diagnosis of pernicious anemia calls for structured follow‐up. First, with the increased risk of gastric adenocarcinoma and Type 1 gastric neuroendocrine tumors, occurring with atrophic gastritis, the American Gastroenterology Association guidelines recommend gastrointestinal endoscopy with topographic biopsy mapping at diagnosis, within approximately 6 months, to confirm atrophic gastritis and exclude prevalent neoplasia [15, 16]. Additional screening for neuroendocrine tumors is recommended at intervals in autoimmune atrophic gastritis [15]. In our patient, an upper gastrointestinal endoscopy and CT scan of the abdomen were scheduled and were deferred due to financial constraints, as these require significant out‐of‐pocket payments. Further, with a possible association with other autoimmune conditions such as Type 1 diabetes, vitiligo, and autoimmune thyroid disease, investigations should exclude these conditions once patients are symptomatic [17, 18]. In our patient, there was no clinical evidence of Type 1 diabetes mellitus, vitiligo, or autoimmune thyroid disease, and so targeted investigations for these conditions were not indicated in the absence of suggestive features. Additionally, structured follow‐up is important to monitor for hypersensitivity to vitamin B12 therapy, although only a few cases have been documented [19]. In our patient, despite markedly elevated levels of serum vitamin B12 acutely following treatment initiation, symptoms suggestive of vitamin B12 toxicity (such as headache, nausea, vomiting, acne, diarrhea, insomnia, anxiety, palpitations, and akathisia) were absent.

Rao (2018) reported the case of a 52‐year‐old vegetarian man who presented with fever, weakness, weight loss, and breathlessness alongside progressive pigmentation, in whom pancytopenia represented an unusual accompanying finding [8]. In that report, the hyperpigmentation involved the palms and axillae, an atypical site for vitamin B12 deficiency [8], in contrast to the more characteristic dorsal hand, foot, and buccal mucosal involvement we observed in our patient. Unlike Rao’s report which does not explicitly describe serial posttreatment hematological or dermatological follow‐up, our case adds longitudinal data at 2 and 4 weeks that helps health practitioners estimate the pace for the normalization of hematological signs and pigmentary resolution following parenteral vitamin B12 therapy initiation.

This case further reinforces that hyperpigmentation and pancytopenia, evaluated together, should raise a clinician’s index of suspicion for vitamin B12 deficiency, particularly among darker‐skinned patients, not dismissing dermatological changes as physiological. Vitamin B12 repletion reverses the hematological and dermatological presentation; however, the more durable clinical task particularly in resource‐constrained settings, is ensuring that the gastric malignancy risk in patients diagnosed with pernicious anemia (autoimmune atrophic gastritis) is continued and not lost to follow‐up once the patient looks and feels well and clinical indices improve.

## Funding

No funding was received for this manuscript.

## Conflicts of Interest

The authors declare no conflicts of interest.

## Data Availability

The data that support the findings of this study are available from the corresponding author upon reasonable request.
